# Germ plasm in *Eleutherodactylus coqui*, a direct developing frog with large eggs

**DOI:** 10.1186/2041-9139-2-20

**Published:** 2011-10-06

**Authors:** Richard P Elinson, Michelle C Sabo, Cara Fisher, Takeshi Yamaguchi, Hidefumi Orii, Kimberly Nath

**Affiliations:** 1 Department of Biological Sciences, Duquesne University, 600 Forbes Avenue, Pittsburgh PA 15282, USA; 2 Laboratory of Regeneration Biology, Graduate School of Life Science, University of Hyogo, 3-2-1 Koto, Kamigori, Akou-gun, Hyogo 678-1297, Japan

## Abstract

**Background:**

RNAs for embryo patterning and for germ cell specification are localized to the vegetal cortex of the oocyte of *Xenopus laevis*. In oocytes of the direct developing frog *Eleutherodactylus coqui*, orthologous RNAs for patterning are not localized, raising the question as to whether RNAs and other components of germ plasm are localized in this species.

**Methods:**

To identify germ plasm, *E. coqui *embryos were stained with DiOC_6_(3) or examined by *in situ *hybridization for *dazl *and *DEADSouth *RNAs. The cDNAs for the *E. coqui *orthologues were cloned by RT-PCR using degenerate primers. To examine activity of the *E. coqui *orthologues, RNAs, made from constructs of their 3'UTRs with *mCherry*, were injected into *X. laevis *embryos.

**Results:**

Both DiOC_6_(3) and *dazl *and *DEADSouth in situs *identified many small islands at the vegetal surface of cleaving *E. coqui *embryos, indicative of germ plasm. *Dazl *was also expressed in primordial germ cells in the genital ridge. The 3'UTRs of *E. coqui dazl *and *DEADSouth *directed primordial germ cell specific protein synthesis in *X. laevis*.

**Conclusions:**

*E. coqui *utilizes germ plasm with RNAs localized to the vegetal cortex to specify primordial germ cells. The large number of germ plasm islands suggests that an increase in the amount of germ plasm was important in the evolution of the large *E. coqui *egg.

## Background

Germ cells are the raison d'être of multicellular organisms, since they are ultimately responsible for the continuation of a species from generation to generation. In many animals, germ cells arise early in development due to a specialized cytoplasmic localization called germ plasm. In other animals, germ cells arise due to interactions between cells, known as inductions. It is curious that such an important cell arises in development in two fundamentally different ways [1,2]. Among amphibians, anurans (frogs) utilize germ plasm, while urodeles (salamanders) utilize inductions [3,4].

Anuran germ plasm contains mitochondria, an electron-dense nuage material, and RNAs. A number of germ plasm specific RNAs have been identified in *Xenopus laevis*, including *dazl*, *nanos1 *(formerly *Xcat2*), *pat*, and *DEADSouth *[5]. During oogenesis, these RNAs are transported to the vegetal cortex of the oocyte. Following fertilization, they are grouped into islands of germ plasm, and as cleavage proceeds the germ plasm islands are segregated into a small number of cells. Cells receiving germ plasm become the primordial germ cells. They migrate from the endoderm into the genital ridges which form the gonads. Orthologues of *dazl *are also localized to germ plasm in the frogs *Lithobates pipiens *and *Pelophylax lessonae*, both formerly placed in the genus *Rana *[6,7].

In addition to the localization of germ plasm RNAs, RNAs involved in patterning the embryo's body are localized to the vegetal cortex. These RNAs in *X. laevis *include *vegt *and *vg1 *[5], and the *vegt *orthologue is also localized in the leopard frog *Lithobates (Rana) pipiens *[6]. The mechanism for transporting the germ plasm RNAs and the patterning RNAs is different [5,8,9]. Germ plasm RNAs accumulate first in the mitochondrial cloud of the small oocyte and then move vegetally via the early METRO pathway. Many patterning RNAs move vegetally via a late pathway.

In contrast, urodeles lack germ plasm [3,10,11] as well as localization of RNAs to the vegetal pole of the oocyte. The latter include the *dazl *orthologue in both *Ambystoma mexicanum *and *Cynops pyrrhogaster *[11,12] as well as the *vegt *orthologue in *A. mexicanum *[13].

The conclusions on RNA localization in germ plasm are based on limited data from only four species, two anurans and two urodeles. Examination of other species, particularly those with diverse life histories, might reveal variations that would indicate how the anuran and urodele patterns arose from their last common ancestor 300 to 250 million years ago (MYA) [14].

In the direct developing frog *Eleutherodactylus coqui*, orthologues of *vegt *and *vg1 *RNAs are not localized to the vegetal cortex [15]. Direct developers like *E. coqui *are derived from anurans with tadpoles. Although the last common ancestor between *E. coqui *and *X. laevis *was 230 MYA, *E. coqui *and *Bufo bufo*, which has germ plasm [16], shared a common ancestor about 53 MYA [14]. *E. coqui *has large 3.5 mm eggs, which show significant differences in embryonic patterning compared to *X. laevis *[17]. These variations raise the question as to whether *E. coqui *has germ plasm with localized RNAs. We examine that question here.

## Methods

### Animals and embryos

*Eleutherodactylus coqui *were collected on the Big Island of Hawaii under Injurious Wildlife Export permits issued by the Department of Land and Natural Resources, Hawaii. Adults were maintained in the laboratory as breeding pairs in terraria as described previously [15,18]. Pairs mated naturally, and clutches of embryos were collected from their guarding father. The embryos were staged according to Townsend and Stewart (TS) [19] and usually cultured in Petri dishes on filter paper, wetted with 20% Steinberg's solution. Ovarian oocytes were obtained by removal from a sacrificed female or by surgical removal from females anesthetized with 0.1% Tricaine methane sulfonate (MS222), made to pH 7.4 by addition of Na_2_HPO_4_. Procedures for *Xenopus laevis *were described previously [20]. Use of *E. coqui *and *X. laevis *in this research was carried out under protocols approved by the Duquesne University Institutional Animal Care and Use Committee (IACUC) and guidelines for animal experiments at the University of Hyogo.

### DiOC_6_(3) staining

Embryos at around the two-cell stage were placed in 20% Steinberg's solution to swell the jelly. The outer jelly layers were removed using watchmaker's forceps and the inner jelly layer was removed by gentle swirling in 3% cysteine, pH8. The green fluorescent dye, DiOC_6_(3) (Invitrogen, Carlsbad CA, USA), was prepared according to Venkatarama *et al*. [21]. The stock, a saturated solution of DiOC_6_(3) in dimethyl sulfoxide, was diluted 2 μl/ml 20% Steinberg's and then further diluted 4 μl/ml 20% Steinberg's. Dejellied embryos were stained for 30 minutes and washed in 20% Steinberg's to remove excess stain. Embryos were prepared for viewing by incubating them in 15% Ficoll in 20% Steinberg's to remove water from the perivitelline space. To view the lower vegetal side, embryos were placed vegetal pole up in a well made of plasticene. The walls of the plasticene well were gently pushed against the inverted embryo to hold it in position. Embryos were viewed with either an Olympus SZx12 stereomicroscope or a Nikon Microphot-SA compound microscope, both equipped with a fluorescent light source. Embryos developed normally after staining and viewing.

### Cloning *E. coqui *orthologues of genes expressed in *X. laevis *germ plasm

To look for RNAs that might identify germ plasm in *E. coqui*, we selected *dazl *and *DEADSouth *(ortholog of *ddx25*). Both RNAs are localized in *X. laevis *germ plasm [5,22,23], and *dazl *orthologue is localized in *Lithobates (Rana) pipiens *and *Pelophylax (Rana) lessonae *germ plasm [5,7]. Our usual cloning route relies on designing degenerate PCR primers based on several orthologous sequences, so we did not try *nanos1*, whose close homologies were only recently recognized, or to *pat*, a germ plasm RNA so far unique to *Xenopus *[24-26]. Vasa is used as a marker for germ plasm in many species, but neither the RNA for the *X. laevis vasa *orthologue nor its protein is localized in *X. laevis *germ plasm [27-29].

RNA was extracted from pieces of ovary with Trizol (Invitrogen) with the addition of a precipitation step with 4 M (final) LiCl at -20C. The RNA was used to make cDNA for PCR amplification of *dazl *and *DEADSouth*. For *dazl*, we started with the degenerate primers designed by Tamori *et al*. [12] for *C. pyrrhogaster*, which yielded 202 nt. An additional round of PCR using an exact primer to the cloned fragment and a degenerate primer, based on *A. mexicanum*, *C. pyrrhogaster*, and *L. pipiens*, generated a 603 nt fragment. The complete cDNA was then obtained from an *E. coqui *ovarian library using exact primers in combination with plasmid primers for T7 or Sp6.

For *DEADSouth*, degenerate primers based on human, mouse, chick, and *X. laevis *yielded a 258 nt fragment. The complete cDNA was obtained from the *E. coqui *ovarian library as above.

### *In situ *hybridization

In order to detect RNA, *in situ *hybridization was carried out on ovarian pieces and on whole embryos as described previously [6,30]. *In situ *hybridization of cleaving embryos proved difficult due their large size and yolkiness, which prevented good fixation. The proteinase K concentration in the procedure was decreased to 5 μg/ml to preserve better the specimens.

### Assaying 3'UTR activity

The 3'UTRs of *E. coqui DEADSouth *(*EcDS3'UTR*) and *dazl *(*EcDazl3'UTR*) were amplified by PCR using a PrimeSTAR enzyme (TaKaRa). They were inserted into the site following the stop codon of *mCherry *of *pCS2-mCherry *without adding any sequence using In-Fusion Advantage PCR Cloning Kit (Clontech). The *mCherry *coding region and *Ec3'UTRs *of *pCS2-mCherry-EcDS3'UTR *and *pCS2-mCherry-EcDazl3'UTR *were confirmed by sequencing. In addition to them, *pCS2-Venus-DEADSouth3'UTR *[20] plasmid was digested with XhoI, and all three constructs were used as templates for *in vitro *mRNA synthesis with mMESSAGE mMACHINE SP6 Kit (Ambion, Austin TX, USA). The mRNAs were dissolved in water, and 4.6 nL of the mRNA (0.1 microgram each mRNA/microliter) was injected into the cortical region at the vegetal pole of a *X. laevis *fertilized egg (460 pg each mRNA/egg). The embryos were allowed to develop at 18°C and were observed at Nieuwkoop - Faber stages 32 and 40 under a Leica MZ16F fluorescent binocular microscope.

## Results

### Migrating primordial germ cells

**Figure 1:**
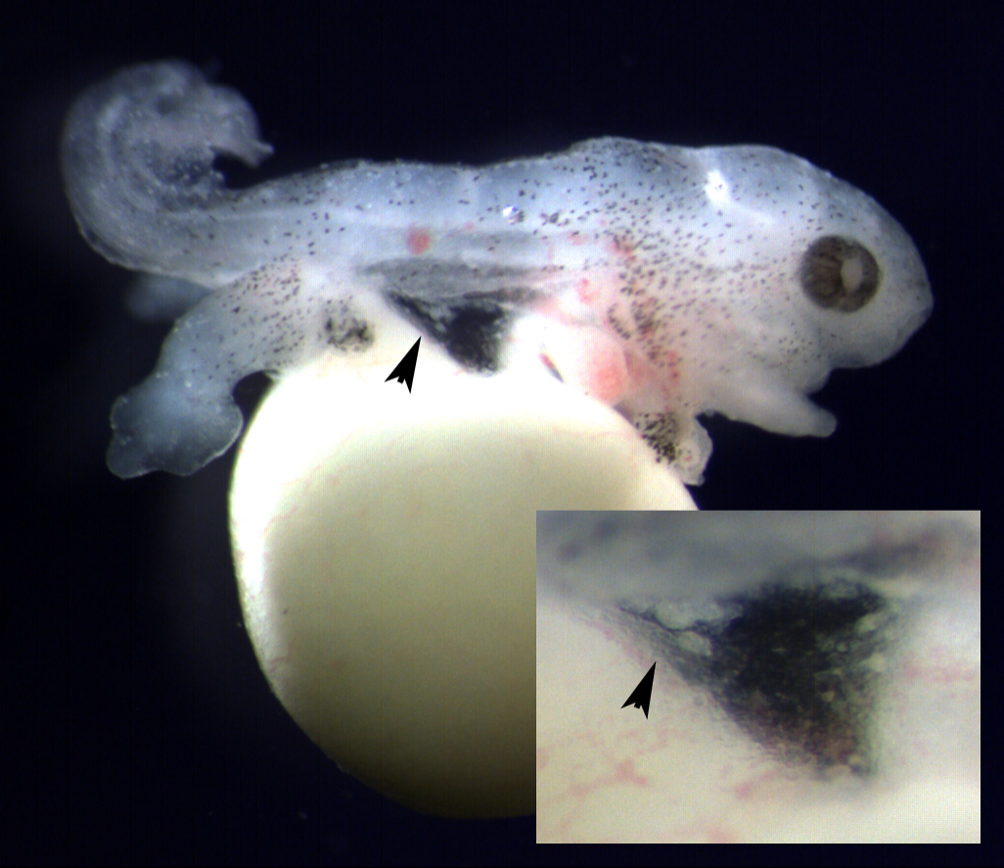
**Migrating PGCs**. The skin was removed from this TS8 embryo to reveal white, yolk-filled PGCs in the posterior part of the dorsal mesentery (arrow). The inset shows an enlargement of this region.

**Figure 2:**
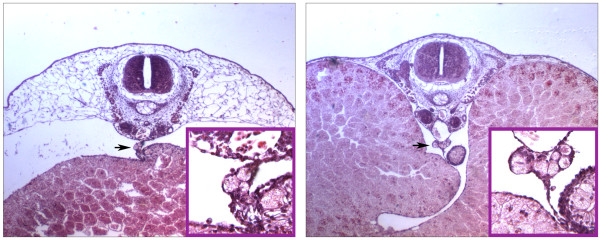
**PGCs in embryo sections**. (A) PGCs are present in the dorsal mesentery (arrow) which attaches the yolky endoderm to the rest of the body of this TS8 embryo. The inset shows an enlargement with three PGCs in the mesentery. (B) At TS9, yolk-filled PGCs are clumped in the prospective genital ridges (arrow), ventral to the dorsal aorta. The inset shows an enlargement of this region.

In several anurans, the primordial germ cells migrate from the endoderm along the dorsal mesentery to the genital ridges [3,16,31,32]. In *X. laevis*, this final migration occurs at Nieuwkoop-Faber stages 41 to 45, prior to tadpole feeding at NF 46 [33,34]. Primordial germ cells, recognizable by their high content of yolk platelets, are visible in the dorsal mesentery in *E. coqui *embryos at Townsend-Stewart (TS) stages 7 to 9 (Figures 1 and 2). They end up near the ventral surface of the dorsal aorta at TS9 (Figures 1 and 2) and reach the genital ridges by TS11.

The presence of *E. coqui *PGCs in the dorsal mesentery suggests that they arise as in other anurans and not as in urodeles. In urodeles, PGCs form by induction in the lateral plate mesoderm, so their migration to the genital ridge is not along the dorsal mesentery [3,4,10,11,35]. If *E. coqui *follow the anuran pattern, they should have localized germ plasm in their oocytes and early embryos. To identify *E. coqui *germ plasm, we used DiOC_6_(3) staining and *in situ *hybridization of orthologues of *dazl *and *DEADSouth*.

### DiOC_6_(3) staining of *E. coqui *cleaving embryos

**Figure 3:**
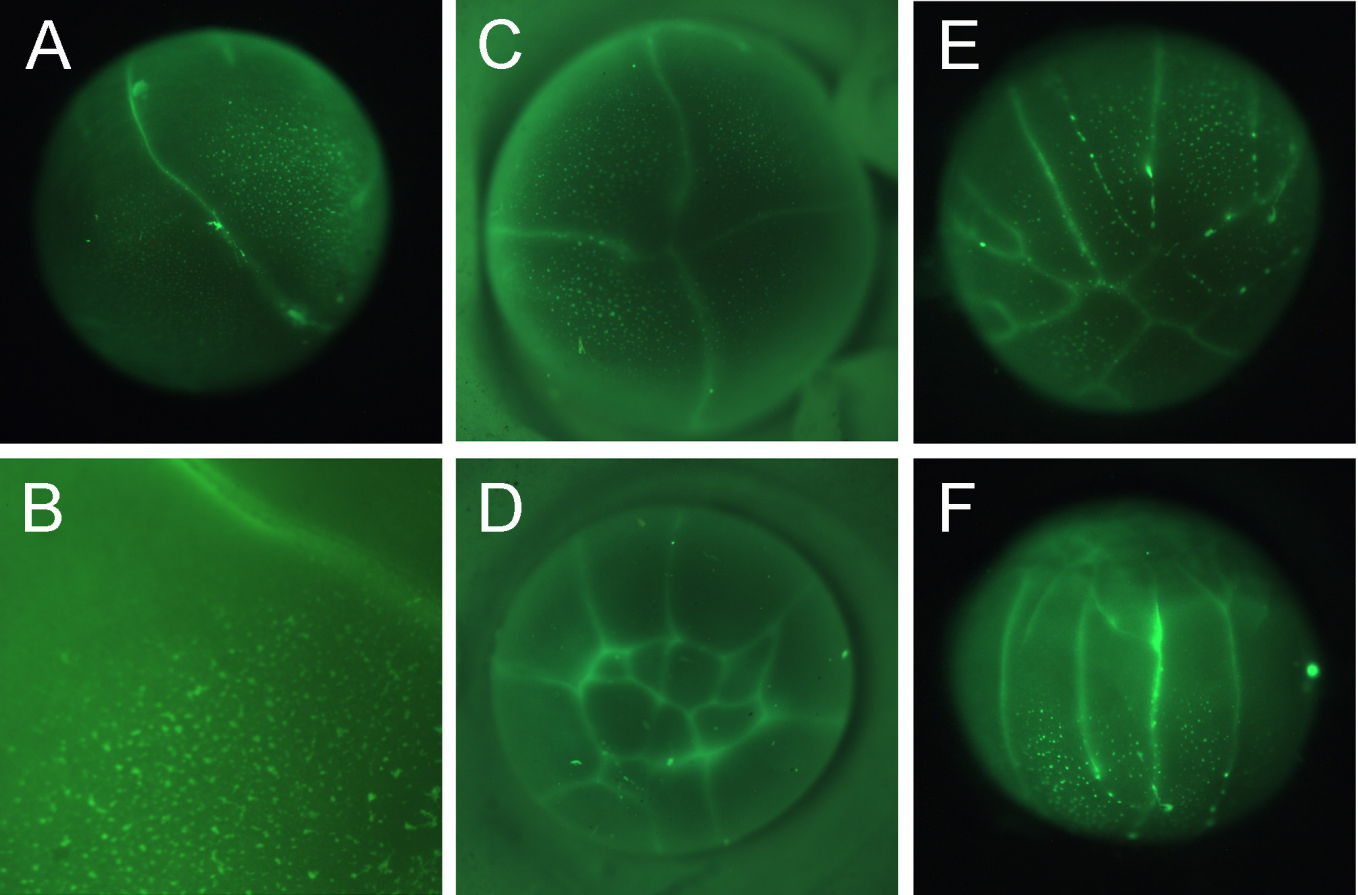
**DiOC_6_(3) staining of cleaving embryos**. (A) Only one furrow (bright green line) has reached the vegetal pole in this eight-cell embryo. A field of small DiOC_6_(3) stained islands is present on the vegetal surface, with most of the islands on one side of the furrow. Furrows stain brightly as they represent two closely-apposed new membranes. (B) This photograph of a different eight-cell embryo shows a cleavage furrow (bright green line) and the boundary between vegetal regions with DiOC_6_(3) stained islands and with no stained islands. (C, D) Vegetal (C) and animal (D) views of the same approximately 16-cell embryo. Although seven cells are present near the animal pole (D), only two furrows are present in the vegetal region (C). DiOC_6_(3) stained islands are present primarily in three of the four vegetal cells (C). (E, F) Vegetal (E) and equatorial (F) views of the same morula stage embryo. Although the region near the animal pole is divided into many cells, a limited number of cleavage furrows have reached the vegetal pole. DiOC_6_(3) islands remain on the vegetal surface, with islands lined up in the forming furrows (E). Embryos are about 3.5 mm in diameter, with (C) enlarged approximately 30% relative to (A) (D) (E) (F).

Germ plasm in *X. laevis *has a high concentration of mitochondria, which allows for the visualization of germ plasm islands in living zygotes using the fluorescent lipophilic dye DiOC_6_(3) [21,36]. When *E. coqui *cleaving embryos were stained with DiOC_6_(3), they showed patterns similar to *X. laevis *germ plasm islands (Figure 3).

The small putative germ plasm islands covered the vegetal third of the two- to eight-cell embryo (Figure 3A, B). At fourth cleavage, there are eight smaller cells near the animal pole (Figure 3D), but cleavage is incomplete at the vegetal pole with two cleavage furrows (Figure 3C). The DiOC_6_(3) islands are usually located asymmetrically relative to the vegetal pole and present in two or three cells (Figure 3C). With continued cleavage, there were fewer germ plasm islands on the surface (Figure 3E, F). Islands accumulated in cleavage furrows (Figure 3E), suggesting as in *X. laevis *that germ plasm leaves the surface by cortical ingression.

### Cloning of *E. coqui *orthologues of *dazl *and *DEADSouth*

To confirm the identification of the DiOC_6_(3) islands in *E. coqui *as germ plasm, we cloned *E. coqui *orthologues of *dazl *and *DEADSouth*, two RNAs localized to germ plasm in *X. laevis*. For convenience, we will refer to these as *Ecdazl *and *EcDEADSouth*. The *Ecdazl *cDNA has 1,698 nucleotides (nt) and contains the complete open reading frame (ORF) for a 267 amino acid protein (GenBank Accession JN130399). There is a 74 nt 5'UTR and a 820 nt 3'UTR. The *Ecdazl *3'UTR is shorter than the 3'UTRs of the orthologues in the frogs *X. laevis *(1,352 nt) and *L. pipiens *(2,635 nt). Nucleotide sequence identities to *dazl *ORFs from the frogs *X. laevis *and *L. pipiens*, the axolotl *Ambystoma mexicanum*, and the mouse *Mus musculus *were 61 to 66%, and amino acid identities were 41 to 45%. These somewhat low identities are true for all pairwise comparisons among these animals with the exception of mouse and axolotl, an anomaly pointed out by Johnson *et al*. [11,37].

Evidence supporting the identification of our clone as *Ecdazl *comes from domain comparisons. Dazl proteins have a conserved RNA Recognition Motif (RRM), which includes two small RNA binding domains RNP-1 and RNP-2. The Ecdazl RRM is 67% identical at the amino acid level to that of *X. laevis*. The seven-amino acid RNP-2 domain is identical between Ecdazl and the four above animals, as well as human, chicken, and a second urodele amphibian *Cynops pyrrhogaster *[12]. The only difference in the seven-amino acid RNP-1 domain among these seven animals is one amino acid difference in *X. laevis *dazl. A recognizable DAZ repeat is present at amino acids 152 to 177 of Ecdazl.

Our cloned *EcDEADSouth *cDNA has 2,024 nt with 38 nt of 5'UTR, a 1,422 nt ORF, and a 564 nt 3'UTR (GenBank Accession JN130398). The ORF is 71% identical to that of *X. laevis *and would code for a protein with 473 amino acids that are 70% identical to *X. laevis *DEADSouth protein. Evidence supporting the identification of our clone as *EcDEADSouth *comes from domain analysis, parallel to that by MacArthur *et al*. [23] for *X. laevis*. The 346-amino acid DEAD-box region is 78% identical and 91% similar to that of *X. laevis*. In one diagnostic sequence, 18/19 amino acids were identical to *X. laevis*. In a second diagnostic sequence of six amino acids, DEADSouth of *X. laevis *shared identity with three amino acids of mouse Mus DEAD5 [23], and those same three amino acids are shared with EcDEADSouth.

The 564 nt 3'UTR is shorter than the 1,597 nt 3'UTR of *X. laevis DEADSouth*, so we tried several approaches to confirm that we have all of the 3'UTR. Our *EcDEADSouth *3'UTR ends in polyA, and numerous attempts to find a longer clone from an *E. coqui *oocyte cDNA library or from cDNA were unsuccessful. There is an AUUAAA sequence which is similar to the polyA signal sequence, although different by one nucleotide. An estimate of the transcript size from Northern blots gave a value slightly over 2 kb, similar to our 2,024 nt sequence. These results indicate that the *EcDEADSouth *3'UTR is significantly shorter than that of *X. laevis*.

### *Ecdazl *and *EcDEADSouth *expression during oogenesis

**Figure 4:**
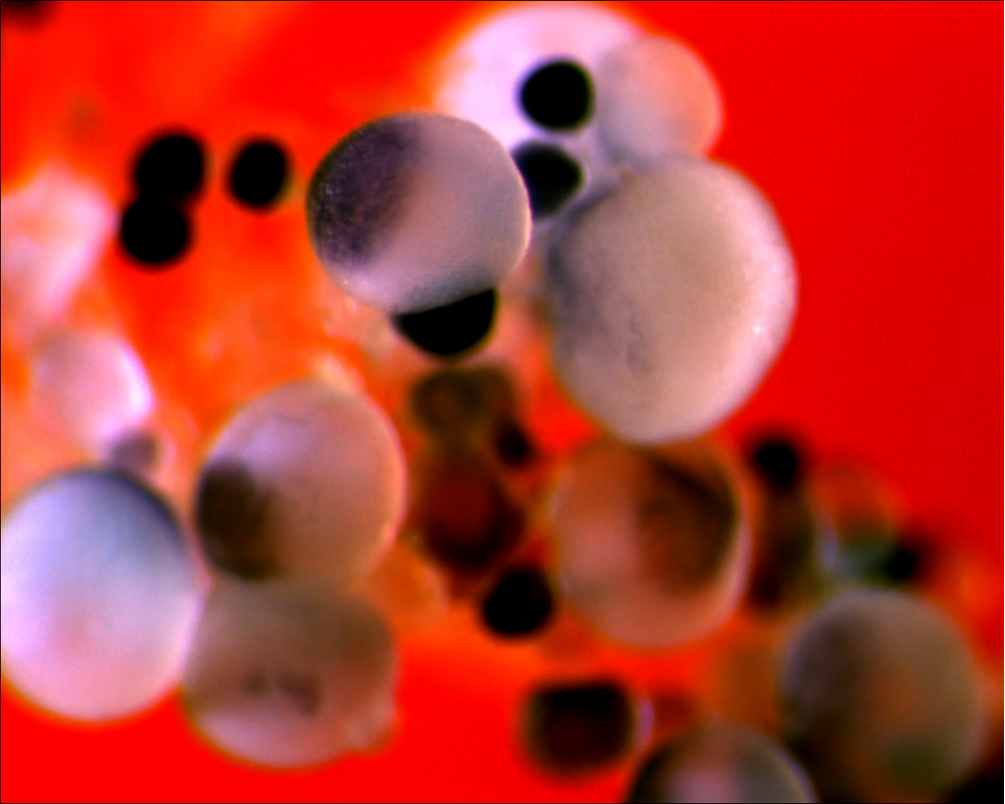
***Ecdazl *expression in oocytes**. In this ovarian fragment, *in situ *hybridization showed *Ecdazl *RNA throughout small oocytes, just beginning vitellogenesis, and localized to one side of vitellogenic oocytes. The vitellogenic oocytes in this preparation are about 1 mm in diameter. Large oocytes, up to 3.5 mm, did not show clear staining and were removed prior to processing of the ovarian fragment.

Pieces of ovary were subjected to whole mount *in situ *hybridization to see whether *Ecdazl *RNA was localized in oocytes, like its orthologues in *X. laevis *and *L. pipiens*. Oocytes, just starting to take up yolk, were stained completely, while small vitellogenic oocytes with yolk showed a clear localization of *Ecdazl *RNA on one side (Figure 4). Similar results were obtained for *EcDEADSouth *RNA. Although *EcDazl *staining asymmetry was present on 1 mm oocytes, there was no obvious staining in larger oocytes.

This lack of staining in large oocytes could be due to the diffuse presence of *Ecdazl *RNA in a large volume. To test this, oocytes were cut into three pieces, animal, equatorial, and vegetal, following the protocol of Beckham *et al*. [15]. RNA was extracted from pooled pieces of each type, and assayed for *Ecdazl *RNA by RT-PCR. Although *Ecdazl *RNA was detected, the results were variable. In two experiments, *Ecdazl *RNA was only in the vegetal third. In a third experiment, the equatorial as well as the vegetal thirds were positive, and in the fourth experiment, *Ecdazl *RNA was found in all three pieces. These results suggest that the location of *Ecdazl *RNA is primarily in the vegetal third, but it may not be as tightly regulated in large oocytes.

### *Ecdazl *and *EcDEADSouth *RNA in cleaving embryos

In order to confirm that the DiOC_6_(3) staining identified germ plasm, we carried out whole mount *in situ *hybridization for *Ecdazl *and *EcDEADSouth *RNAs on cleaving embryos. Good fixation of these large, yolky cleaving embryos was difficult, and the vegetal surface tended to be lost in the many changes of solutions. Nonetheless, the *in situ *patterns were similar to the patterns found with DiOC_6_(3) staining.

**Figure 5:**
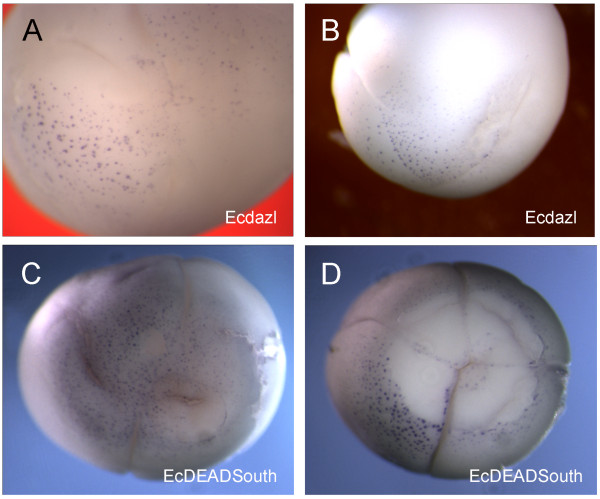
***Ecdazl *and *EcDEADSouth *RNA in cleaving embryos**. (A) In this vegetal view, many islands containing *Ecdazl *RNA are visible near the surface of a four-cell embryo. Cleavage furrows have not reached the vegetal half. (B) In this side view of a 16-cell embryo, islands of *Ecdazl *RNA are present near the vegetal pole. Cleavage furrows have reached the vegetal half. (C) In this vegetal view, many *EcDEADSouth *islands are visible despite some surface damage. (D) In this 16-cell embryo with two furrows reaching the vegetal pole, most of the surface surrounding the vegetal pole has been lost, revealing the whiter underlying cytoplasm. The surface loss permits several observations. First, *EcDEADSouth *islands are visible on the intact surface. Second, the islands are located asymmetrically relative to the vegetal pole, marked by the intersecting furrows. Third, *EcDEADSouth *islands are present in the cleavage furrow, where internal cytoplasm is exposed.

For both *Ecdazl *and *EcDEADSouth*, there were many small stained islands on the vegetal surface of four to eight cell embryos (Figure 5). At 16 to 32 cells, the islands were usually displaced to one side of the vegetal pole (Figure 5B, D). As with DiOC_6_(3), the RNAs were present within cleavage furrows (Figure 5B, D). Based on the similarity of patterns, we conclude that staining with DiOC_6_(3), *Ecdazl*, and *EcDEADSouth *identifies germ plasm near the vegetal surface of *E. coqui *cleaving embryos.

### *Ecdazl *RNA in primordial germ cells

**Figure 6:**
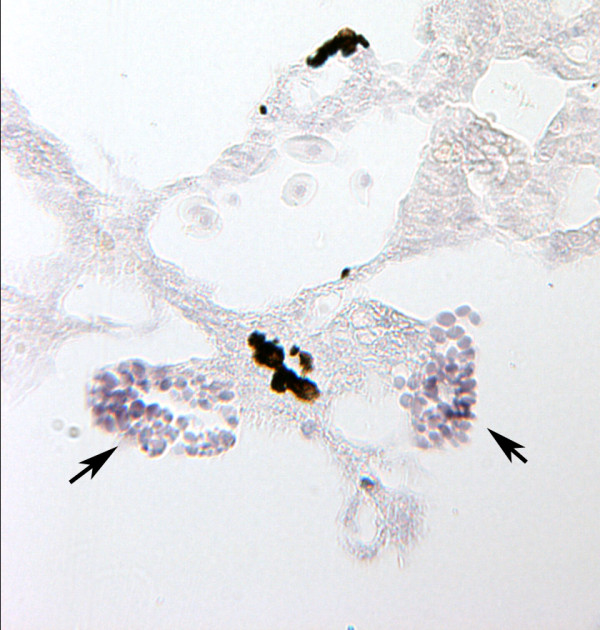
***Ecdazl *expression in primordial germ cells**. Yolk-filled cells with *Ecdazl *RNA (arrows) are present in the genital ridges of this TS14 embryo.

We have not attempted to track the fate of cells with germ plasm in these large embryos after the cleavage stages, but we carried out *in situ *hybridization on sections of TS14 and TS15 embryos to see whether the primordial germ cells in the genital ridges expressed *Ecdazl*. TS15 is the stage at which the froglet hatches from its jelly capsule and prior to feeding. Yolk-filled cells in the genital ridges stained positively with the *Ecdazl *probe (Figure 6). This early gonadal expression of *Ecdazl *contrasts with the pattern in *X. laevis *or *Xenopus tropicalis*. In those frogs, *dazl *RNAs disappear before their tadpoles begin feeding, but the genes are re-expressed in the frog testis and ovary [22,38]. Xdazl protein, however, remains in the primordial germ cells of tadpoles [20,39]. Since the tadpole was deleted in the evolution of direct development, it is expected that the gonad of a TS15 *E. coqui *froglet would be more equivalent to a gonad in newly metamorphosed *Xenopus *than to its tadpole.

### Activity of *EcDEADSouth *and *Ecdazl *3'UTRs

The 3'UTRs of *DEADSouth *and *dazl *are sufficient to program translation of an attached ORF in the primordial germ cells of *X. laevis *[20,40]. This raises the question as to whether the 3'UTRs of *EcDEADSouth *and *Ecdazl *would have activity in this assay. Both of the *E. coqui *3'UTRs are much shorter than the *X. laevis *orthologues, and there is little overall nucleotide sequence identity.

**Figure 7:**
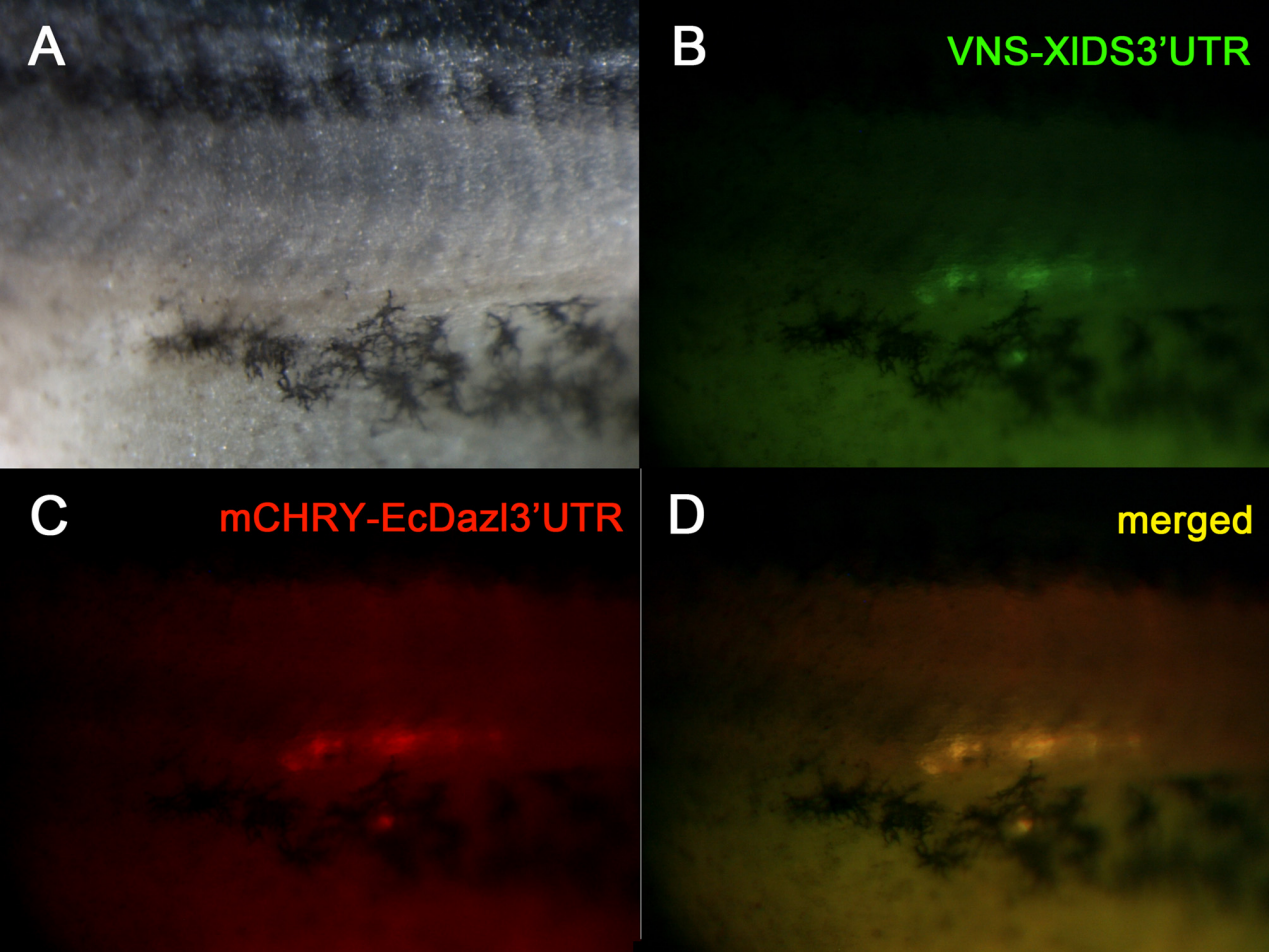
**Activity of the *EcDazl3'UTR***. A *X. laevis *egg was injected with RNAs of both *Venus-XlDS3'UTR *and *mCHERRY-EcDazl3'UTR*. The genital ridge region of the embryo (A) was examined at stage 40 with different light sources to detect Venus (B, green) and mCHERRY (C, red) proteins. The merged image (D, yellow) shows that these two proteins co-localized. The co-localization indicates that the *EcDazl 3'UTR *directed PGC-specific protein expression in *X. laevis*.

To test their activities, the 3'UTRs were placed in plasmids with *mCherry*, and RNAs were synthesized *in vitro*. Either the *mCherry-EcDS3'UTR *RNA or the *mCherry-EcDazl3'UTR *RNA was microinjected into *X. laevis *fertilized eggs along with *Venus-XlDS3'UTR *RNA. The *X. laevis DEADSouth 3'UTR *led to synthesis of Venus protein in PGCs, and mCherry protein co-localized with Venus using *3'UTRs *of either *EcDEADSouth *(10/10) or *Ecdazl *(6/7) (Figure 7).

## Discussion

### Large surface area covered by islands of germ plasm in *E. coqui*

Our present results indicate that *E. coqui*, with big 3.5 mm eggs, has germ plasm similar to frogs such as *X. laevis*, with 1.3 mm eggs, and *L. pipiens*, with 1.7 mm eggs. Islands of *E. coqui *germ plasm were identified by DiOC_6_(3) staining and by localization of *Ecdazl *and *EcDEADSouth *RNAs, and they are spread over a large part of the vegetal cortex of the cleaving embryo. Their distribution is similar to that found in *X. laevis*, but the *E. coqui *germ plasm covers a more extensive area both relatively and absolutely.

In relative terms, germ plasm in *X. laevis *becomes concentrated near the vegetal pole by the time the second cleavage furrow reaches that pole at the four- to eight-cell stage [22,23,25,36,38,41-43]. Germ plasm is removed from the surface along the furrows by cortical ingression [36,41], so that little remains by 32 cells. In contrast, many islands of germ plasm remain at the surface of *E. coqui *morulae. *E. coqui *germ plasm also leaves the surface along cleavage furrows, but the furrows arrive later and the vegetal cells remain larger compared to *X. laevis*. This less active early removal is coupled with the greater extent of surface coverage by germ plasm in *E. coqui*.

In absolute terms, *E. coqui *germ plasm extends far towards the equator from the vegetal pole. If we estimate that germ plasm is present from the pole to the 45° latitude, that surface area is 5.8 mm^2^, which is greater than the surface area of an entire *X. laevis *egg (5.3 mm^2^).

An important function of germ plasm in *X. laevis *is to repress transcription, thereby preventing primordial germ cells from responding to inductive signals from neighboring cells [21]. Transcriptional repression by germ plasm has also been documented in *Drosophila *and *C. elegans *[44], indicating that this activity may be the main role for germ plasm in the early embryo. If *E. coqui *has a larger area covered by germ plasm, how can it avoid the negative consequences of transcriptional repression in many vegetal cells?

One possibility is that transcriptionally repressed vegetal cells can be tolerated in *E. coqui *because most vegetal cells serve only a nutritional function and do not contribute to any embryonic tissues. We previously defined these cells as nutritional endoderm [45]. Direct development is a derived condition resulting from the evolutionary loss of the aquatic-feeding tadpole from the life history. To compensate for the lack of a feeding larva, the oocyte was provisioned with more yolk and increased in size. Increased egg size, however, could pose difficulties for the migration of primordial germ cells from the vegetal pole to their target, the genital ridge. One solution would be to increase the number of potential primordial germ cells, making it probable that some would be in position to migrate out of the endoderm and reach the genital ridges. While this is a speculative hypothesis to account for the large surface covered by germ plasm, a testable prediction is that there will be cells with germ plasm trapped among the cells of the nutritional endoderm at later stages. These cells will be lost along with the yolk-depleted cells of the nutritional endoderm.

Alternatively, the presence of transcriptional repressors in a greater number of vegetal cells may be part of the mechanism generating nutritional endoderm. Cells of the nutritional endoderm appear undifferentiated. While this may be due to the lack of signaling by nodal or other ligands [17], transcriptional repression could ensure that these cells remain in an embryonic state as their yolk is depleted. Venkatarama *et al*. [21] showed by immunocytochemistry that transcriptional elongation occurred in early somatic cells but not in primordial germ cells. A similar immunocytochemical approach would provide a test of whether repression of transcriptional elongation is widespread in the nutritional endoderm.

### Evolutionary differences in RNA localization in amphibian oocytes

In *X. laevis*, there are three groups of RNAs, localized to the vegetal cortex of the oocyte, that are necessary for embryogenesis. RNAs for germ plasm are localized to the vegetal cortex during oogenesis via the early or METRO pathway, while RNAs involved in germ layer specification, notably *vegt*, are localized via the late pathway [5,8]. The third group of localized RNAs is involved in establishing the embryo's dorsal axis, which is specified by the microtubule-dependent cortical rotation in the first cell cycle after fertilization [46,47]. These RNAs include *wnt11*, *fatvg *(*plin2*), and *trim36*. *Wnt11 *plays a major role in causing movement of β-catenin into nuclei on the dorsal side [48-50]. Both *fatvg *and *trim36 *are required for the cortical rotation, and the parallel array of microtubules does not form in the absence of *trim36 *[51,52]. All three of these RNAs are localized to the vegetal cortex via the early pathway, although there is some movement of *fatvg *RNA via the late pathway [51].

The localization of *Ecdazl *and *EcDEADSouth *to the vegetal cortex of *E. coqui *small oocytes suggests the presence of the early localization pathway. Prior to localization, however, these RNAs were detected throughout the oocyte and not localized to an organelle, such as the mitochondrial cloud or Balbiani body as in *X. laevis *[53,54]. Attempts to visualize a Balbiani body in *E. coqui *oocytes have not been successful. Organelle localization of a *dazl *orthologue was also not found in *Lithobates pipiens *and *Pelophylax lessonae*, both formerly placed in the genus *Rana *[6,7].

Although the early RNA localization pathway appears to exist in *E. coqui*, the late localization pathway does not. Neither *vegt *nor *vg1 *RNA, are localized to the vegetal cortex, and these orthologous RNAs are present diffusely in the animal cytoplasm [15]. Their animal location correlates with the more animal origin of both mesoderm and definitive endoderm in *E. coqui *[17,45].

The presence of RNAs localized to germ plasm in *E. coqui *raises the question as to the presence of maternal RNAs for specification of the dorsal axis. Although a cortical rotation has not been documented for *E. coqui*, it likely exists. There is a massive vegetal array of parallel microtubules which persists beyond first cleavage, and artificially moving cytoplasm relative to the cortex by tilting zygotes 90° with respect to gravity can specify the dorsal axis [55]. In the current work, islands of germ plasm usually extended closer to the equator on one side of the cleaving embryo, suggesting that a cortical rotation occurred and caused this asymmetry. It would be interesting to observe the behavior of *wnt11 *RNA in *E. coqui*, with the predictions that it would be localized to the vegetal cortex in oocytes and that it would be shifted to one side equatorially during cleavage.

In the urodele *A. mexicanum*, the orthologous *vegt *RNA is not localized [13], raising the possibility that as in *E. coqui*, the late pathway is absent. Urodeles lack germ plasm and its associated RNAs, suggesting that the early pathway also is absent. Nonetheless, specification of the dorsal axis in urodeles is likely caused by a cortical rotation. A grey crescent, which forms due to the rotation, appears after fertilization in several species [56-58], and cortical arrays of parallel microtubules have been detected in *C. pyrrhogaster *[59]. These observations raise the question as to whether RNA of orthologues of *wnt11*, *fatvg*, and *trim 36 *are localized in urodele oocytes or whether urodeles use a different mechanism to initiate development of the dorsal axis.

### Conservation of PGC RNA behavior

The last common ancestor of *X. laevis *and *E. coqui *lived about 230 MYA. Although the 3'UTR nucleotide sequences of the *DEADSouth *and *dazl *orthologues are very different, the activity of the 3'UTRs in directing PGC specific translation has been conserved apparently over this long period of time. Unlike the constraints on sequence changes within an ORF, many 3'UTR functions require only short sequences. These sequences could be stems for generating secondary RNA structures, recognized by a protein, or they could be targets for microRNA binding.

Two microRNAs have been implicated in control of RNAs in *X. laevis *PGCs, namely *miR-427 *and *miR-18 *[40,60]. *miR-427 *mediates the destruction of many maternal RNAs [60] and is the orthologue of zebrafish *miR-430*, which is involved in regulation of RNAs in zebrafish PGCs [61-63]. *miR-18 *causes the destruction of the *Dead end *RNA in somatic cells, but *Dead end *RNA is protected in the PGCs [40]. Accordingly, we examined the 3'UTRs of the *DEADSouth *and *dazl *orthologues for target sites for these and other miRs [64]. The 3'UTR of *X. laevis DEADSouth *has four putative targets of *miR427*, *EcDEADSouth *has one, *X. laevis dazl *has one, and *Ecdazl *has two. The 3'UTR of *X. laevis DEADSouth *has two putative targets of *miR-18*, but no *miR-18 *targets were present in the 3'UTRs of *EcDEADSouth*, *Ecdazl*, or *X. laevis dazl*. These comparisons suggest that *miR-427 *should be the first candidate examined to account for the conservation of 3'UTR activities between *X. laevis *and *E. coqui*.

## Conclusions

The great increase in yolk in the evolution of direct development in *E. coqui *has led to changes in the organization of its egg and early development. Compared to *X. laevis *and most anurans with small eggs and aquatic development, the changes include: slow and delayed cleavage of the vegetal region (Figure 3), lack of localization to the vegetal cortex of RNAs involved in germ layer patterning [15], and the origin of the novel nutritional endoderm [18,45]. Despite these profound changes of the yolk-rich vegetal region, formation of primordial germ cells via germ plasm, localized to the vegetal cortex, has been conserved. These direct developers have not moved towards an alternative path to generate primordial germ cells, such as found in urodeles. Rather, they appear to have expanded the relative amount of germ plasm coverage of the vegetal region. Potential reasons for this expansion of germ plasm are to participate in the formation of the nutritional endoderm and to ensure that some primordial germ cells, among the huge number of vegetal cells, get to the genital ridge. These hypotheses require future experimental testing.

## Abbreviations

IACUC: Institutional Animal Care and Use Committee; nt: nucleotides; ORF: open reading frame.

## Competing interests

The authors declare that they have no competing interests.

## Authors' contributions

RPE designed the study, carried out the observations with DiOC_6_(3), and drafted the manuscript. MCS and CF cloned the first pieces of *Ecdazl *and *EcDEADSouth *cDNA, respectively, which they began as undergraduate project students. TY and HO prepared the 3'UTR constructs and carried out the assay of their activity. KN obtained complete clones of *Ecdazl *and *EcDEADSouth *cDNA, analyzed their sequences, and carried out the *in situ *hybridizations. All authors read and approved the manuscript.
